# Effect of Liraglutide on Cardiac Function in Individuals With Type 2 Diabetes: A Meta-Analysis

**DOI:** 10.7759/cureus.42651

**Published:** 2023-07-29

**Authors:** Hasnaat Haroon, Ajanta Kumari, Bihari Lal, Ankeeta Kumari, Jasvant Kumar, Khaldoun Khreis, Majed Sheikh, Adil Amin

**Affiliations:** 1 Internal Medicine, Medical College, Foundation University Medical College, Islamabad, PAK; 2 Internal Medicine, Jinnah Sindh Medical University, Karachi, PAK; 3 Internal Medicine, Chandka Medical College Larkana, Larkana, PAK; 4 Medicine, Liaquat University of Medical and Health Sciences, Jamshoro, Karachi, PAK; 5 Internal Medicine, Chandka Medical College Larkana, Jacobabad, PAK; 6 Pediatrics, Pécs Medical University, Pécs, HUN; 7 Cardiology, Royal Free London Foundation Trust, London, GBR; 8 Cardiology, Pakistan Navy Ship (PNS) Shifa, Karachi, PAK

**Keywords:** meta-analysis, type 2 diabetes, diastolic cardiac function, systolic cardiac function, liraglutide

## Abstract

The aim of this study was to determine the effect of liraglutide on cardiac function in individuals with type 2 diabetes. The present meta-analysis aimed to identify studies testing liraglutide in individuals with type 2 diabetes. We included observational and randomized controlled trials comparing liraglutide with placebo or any other drug alone or in combination with other drugs. A comprehensive search was carried out using online databases including PubMed, Google Scholar, and Cochrane Library to find relevant studies from inception to June 30, 2023, according to the Preferred Reporting Items for Systematic Reviews and Meta-Analysis (PRISMA) guidelines. Key terms used to search for relevant studies included "liraglutide," "cardiac function," and "type 2 diabetes," along with their synonyms and Medical Subject Heading (MeSH) terms. The outcomes assessed in the present meta-analysis included diastolic cardiac function and systolic cardiac function. For diastolic cardiac function, we assessed the E to A (E/A) ratio and the E to Ea (E/Ea) ratio. To assess the impact of liraglutide on systolic function, we assessed stroke volume in mL, left ventricular ejection fraction (LVEF) in %, cardiac output in L/min, and cardiac index in L/min/m². A total of seven studies were included, with a pooled sample size of 307 individuals (160 in the liraglutide group and 147 in the control group). The results indicated that liraglutide significantly reduced the E/A ratio (mean difference [MD]: -0.22, 95% CI: -0.38 to -0.06, p-value: 0.008) and E/Ea ratio (MD: -0.76, 95% confidence interval (CI): -1.39 to -0.12, p-value: 0.02, suggesting a potential clinical benefit on ventricular diastolic function. However, there was no significant impact on LVEF (MD: 0.46, 95% CI: -3.13 to 4.05, p-value: 0.80), cardiac output (MD: 0.05, 95% CI: -0.39 to 0.49), cardiac index (MD: 0.07, 95% CI: -0.18 to 0.32), and stroke volume (MD: -5.34, 95% CI: -14.81 to 4.12), indicating that liraglutide did not improve systolic function.

## Introduction and background

Type 2 diabetes is a complicated metabolic disorder characterized by high blood sugar levels and is associated with an increased risk of cardiovascular (CV), microvascular, and other complications [1]. Individuals with type 2 diabetes have a two to five times higher likelihood of developing heart failure [2]. Diabetic cardiomyopathy, which involves impaired relaxation of the left ventricle (LV), can progress to heart failure with preserved ejection fraction (HFpEF) [2]. Most patients with diabetic cardiomyopathy who experience heart failure symptoms are classified as having HFpEF. HFpEF poses a significant risk of morbidity [3] and mortality [4] for patients with type 2 diabetes. Therefore, early detection and medical treatment to reverse LV diastolic dysfunction are crucial in managing diabetes. Although strict control of blood sugar levels is essential in reducing diabetes-related complications, it alone does not appear to improve LV diastolic function [5].

Liraglutide is an approved glucagon-like peptide 1 receptor agonist (GLP-1RA) used in the treatment of type 2 diabetes. Apart from reducing glucose levels, GLP-1RA also offers other benefits such as weight loss and a low risk of hypoglycemia. In addition to its glycemic effects, GLP-1RA has gained attention due to its potential beneficial impact on CV function [6]. Liraglutide, an anti-diabetic agent, is a GLP-1RA that enhances insulin secretion, suppresses glucagon production, and promotes weight loss. While some studies have explored the effects of GLP-1RA on ischemic heart disease and heart failure with reduced ejection fraction (HFrEF) [7], little is known about their impact on LV diastolic function. Weight loss induced by GLP-1RA treatment may improve LV diastolic function [8]. Moreover, preclinical studies and certain human studies suggest a direct cardioprotective effect of GLP-1RA therapy [9].

Diastolic heart failure is common in individuals with type 2 diabetes and occurs before overt heart failure [10]. Detecting diastolic heart failure may help identify high-risk individuals who would benefit from early and more aggressive intervention to prevent the development of overt heart failure [10]. Several small clinical studies have demonstrated positive effects on systolic heart function following GLP-1 treatment [11-12]; however, recent studies have been inconclusive [13-14]. Nevertheless, there is a scarcity of studies on liraglutide treatment in patients with type 2 diabetes [15]. Therefore, we are conducting a pooled analysis to determine the effect of liraglutide on cardiac function in individuals with type 2 diabetes.

## Review

Methodology

This meta-analysis was reported according to the Preferred Reporting Items for Systematic Reviews and Meta-Analysis (PRISMA) guidelines. The protocol of this was registered with PROSPERO with registration number CRD42023413995. The present meta-analysis aimed to identify studies testing liraglutide in individuals with type 2 diabetes. We included observational and randomized controlled trials (RCTs) comparing liraglutide with placebo or any other drug alone or in combination with other drugs. Secondly, the outcomes of the included studies must contain the outcomes assessed in the current study. We excluded case reports, reviews, crossover studies, and studies lacking a control group.

Search Strategy

A comprehensive search was carried out using online databases including PubMed, Cochrane Library, and Google Scholar to find relevant studies from inception to June 30, 2023, according to the PRISMA guidelines. Key terms used to search for relevant studies included "liraglutide," "cardiac function," and "type 2 diabetes," along with their synonyms and Medical Subject Heading (MeSH) terms. We used Boolean operators (AND, OR) to further refine the search (as shown in the Appendix). The reference lists of all included studies were manually searched for additional studies. Study searching was performed independently by two authors. Any disagreement in this process was resolved through discussion.

Data Extraction and Quality Assessment

Two authors independently screened all eligible studies using their titles and abstracts to determine whether they were eligible to be included in this meta-analysis. The full text of all eligible records was obtained, and a detailed assessment was conducted based on predefined inclusion and exclusion criteria.

Data from included studies were extracted using Microsoft Excel Spreadsheet. The extracted data included the first author's name, year of publication, study design, groups, dose, sample size, follow-up duration, and patients' characteristics. One author extracted the data, and the second author cross-checked and entered it into the Review Manager (RevMan) software (version 5.4.1, The Cochrane Collaboration, London, United Kingdom) for analysis. Quality assessment of all included studies was performed using the Cochrane Risk of Bias Assessment tool for RCTs [16] and the Newcastle-Ottawa Scale for observational studies [17]. Any disagreement in the process of study selection, data extraction, and quality assessment was resolved through discussion.

Outcome Measures and Data Analysis

The outcomes assessed in the present meta-analysis included diastolic cardiac function and systolic cardiac function. For diastolic cardiac function, we assessed the E to A ratio (E/A) and the E to Ea ratio (E/Ea). To assess the impact of liraglutide on systolic function, we assessed stroke volume in mL, left ventricular ejection fraction (LVEF) in %, cardiac output in L/min, and cardiac index in L/min/m^2^.

The data analysis for this study was conducted using the RevMan software. The primary outcome measure of interest was the mean difference between groups, accompanied by its 95% confidence interval (CI). A p-value cutoff of less than 0.05 was set to determine statistical significance. To assess heterogeneity among the included studies, the I-square (I²) statistic was employed. An I² value greater than 50% was considered significant, indicating significant heterogeneity, and a random-effects model was used. Otherwise, a fixed-effect model was used.

Results

**Figure 1:**
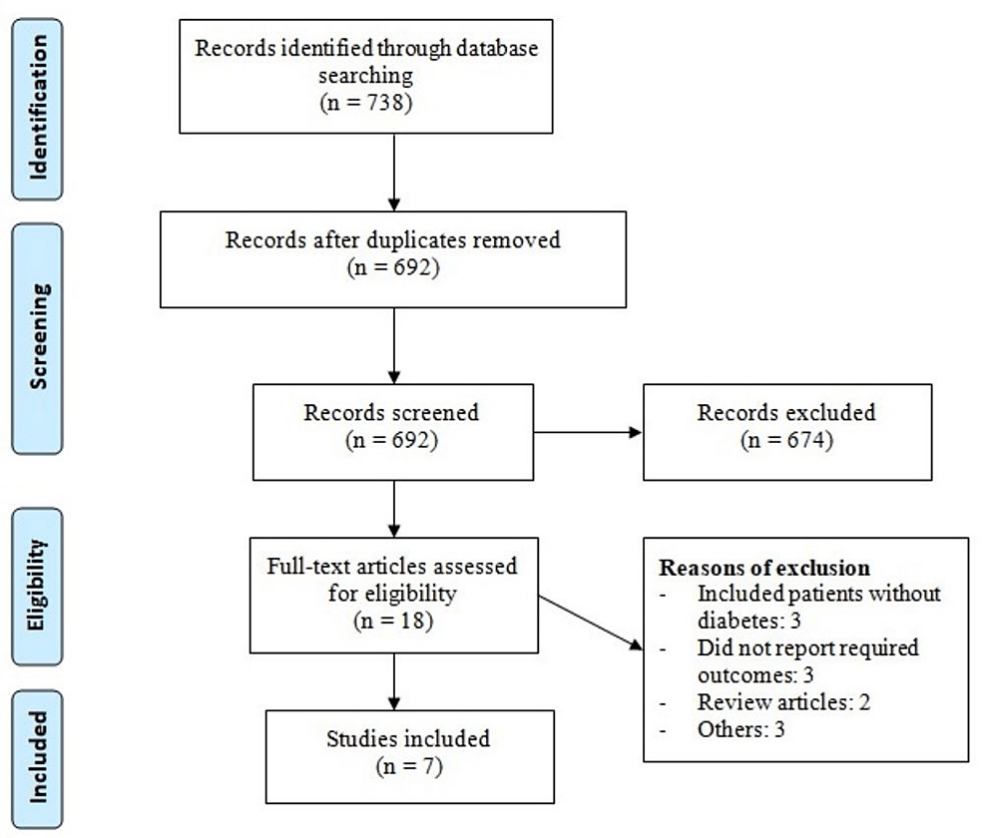
PRISMA flowchart of study selection PRISMA, Preferred Reporting Items for Systematic Reviews and Meta-Analysis

**Figure 2:**
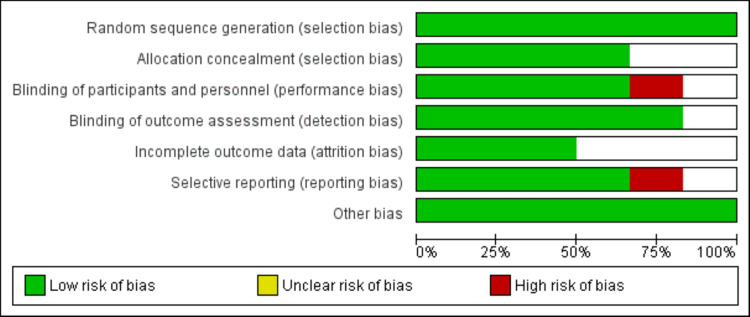
Risk-of-bias assessment

Online database searching yielded 738 studies. After removing duplicates, 692 studies were initially screened using their titles and abstracts. Eighteen studies were eligible for full-text screening. Based on the detailed assessment of these records, seven studies were included in the final analysis. Figure 1 shows the process of study selection. Table 1 shows the characteristics of included studies. Pooled sample size of this meta-analysis was 307 individuals (160 in the liraglutide group and 147 in the control group). The follow-up of included studies ranged from 16 weeks to 52 weeks. Two studies included patients with shistory of heart failure [6,18]. Majority of participants were males. Figure 2 presents the risk-of-bias assessment of the included studies. Majority of the studies were double-blinded. Overall, the quality of studies was high.

**Table 1:** Characteristics of included studies RC, retrospective cohort; RCT, randomized controlled trial

Author Name	Year	Study Design	Study Groups	Dose of Liraglutide	Sample Size	Follow-Up	Mean Age (Years)	Male (%)	Heart Failure
Arturi et al. [18]	2016	RCT	Liraglutide	1.8 mg/day	10	52 weeks	59.5 vs 60	70 vs 90	100%
			Control		10				
Bizino et al. [19]	2019	RCT	Liraglutide	1.8 mg/day	23	26 weeks	60 vs 59	61 vs 58	0%
			Control		26				
Jørgensen et al. [15]	2016	RCT	Liraglutide	1.8 mg/day	16	16 weeks	NR	NR	0%
			Control		16				
Lambadiari et al. [20]	2018	RCT	Liraglutide	1.8 mg/day	30	26 weeks	51 vs 50	66.7 vs 66.7	NR
			Control		30				
Nyström et al. [6]	2017	RCT	Liraglutide	1.8 mg/day	33	18 weeks	61 vs 63	72.7 vs 72.4	100%
			Control		29				
Paiman et al. [21]	2019	RCT	Liraglutide	1.8 mg/day	22	26 weeks	55 vs 55	36 vs 44	0%
			Control		25				
Saponaro et al. [22]	2016	RC	Liraglutide	0.6 to 1.8 mg/day	26	26 weeks	61.7 vs 63.5	58.7 vs 63.6	0%
			Control		11				

Meta-analysis of outcomes

Diastolic Function

**Figure 3:**
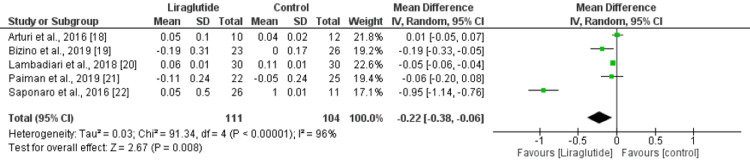
Change in E/A from baseline Sources: [18-22]

**Figure 4:**
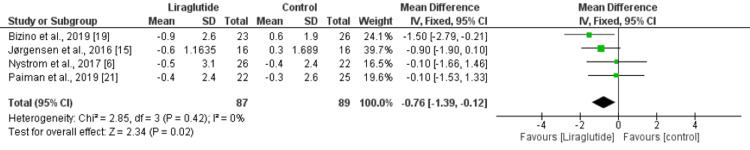
Change in E/Ea from baseline Sources: [6,15,19,21]

We used the E/A ratio and the E/Ea ratio to assess the impact of liraglutide on diastolic function in patients with type 2 diabetes. The effect of liraglutide on E/A ratio was presented in five studies enrolling 215 patients. Pooled analysis showed that reduction in E/A ratio was significantly greater in the liraglutide group compared to the control group (mean difference [MD]: -0.22, 95% CI: -0.38 to -0.06, p-value: 0.008). High heterogeneity was reported among the study results. We performed sensitivity analysis by removing the study conducted by Saponaro et al. [22]. As a result, heterogeneity was reduced to 46% without affecting the relationship between liraglutide and E/A ratio (MD: -0.05, 95% CI: -0.10 to -0.01) (Figure 3). Regarding the E/Ea ratio, four studies were included in the pooled analysis of liraglutide enrolling 176 patients with type 2 diabetes. As shown in Figure 4, reduction in the E/Ea ratio was significantly greater in patients in the liraglutide group compared to the control group (MD: -0.76, 95% CI: -1.39 to -0.12, p-value: 0.02). No significant heterogeneity was reported among the study results.

Systolic Function

**Figure 5:**
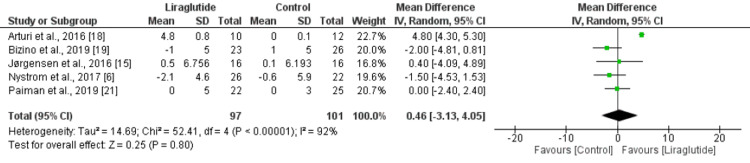
Change in LVEF (%) from baseline Sources: [6,15,18-19,21] LVEF, left ventricular ejection fraction

The effect of liraglutide on LVEF (%) is shown in Figure 5. Pooled analysis of five studies showed no significant difference between the two groups in terms of change in LVEF from baseline (MD: 0.46, 95% CI: -3.13 to 4.05, p-value: 0.80). High heterogeneity was reported among the study results. We performed sensitivity analysis by excluding studies that included patients with heart failure [6,18]. After removing these studies, the heterogeneity was reduced to 0%. However, the effect of liraglutide on change in LVEF remained insignificant (MD: -0.67, 95% CI: -2.36 to 1.02).

The effect of liraglutide on other measures of systolic function including cardiac output, cardiac index, and stroke volume is shown in Table 2. Pooled analysis did not report any significant difference in any of the aforementioned systolic function indicators between patients in the liraglutide group and the control group.

**Table 2:** Systolic function outcomes MD, mean difference; CI, confidence interval

Outcomes	MD (95% CI)	I^2^
Cardiac output (mL)	0.05 (-0.39 to 0.49)	86%
Cardiac index (L/min/m^2^)	0.07 (-0.18 to 0.32)	86%
Stroke volume (L/min)	-5.34 (-14.81 to 4.12)	94%

Discussion

In the present meta-analysis, we assessed the effect of liraglutide on cardiac function in individuals with type 2 diabetes. The pooled results showed that liraglutide significantly reduced the E/A ratio and the E/Ea ratio, but had no significant impact on LVEF, cardiac output, cardiac index, and stroke volume. These results show that liraglutide does not improve systolic function but may have potential clinical benefits on ventricular diastolic function. While a recent study with a placebo-controlled crossover design did not find any improvement in systolic function among newly diagnosed patients with type 2 diabetes treated with liraglutide [23], there is some evidence suggesting a potential role of this drug in improving diastolic heart function and cardiac remodeling [24-25]. Another prospective observational study in patients with type 2 diabetes demonstrated that six months of liraglutide treatment led to a significant enhancement in diastolic function alongside body weight reduction [26]. However, it is worth noting that this study had a major weakness in terms of lacking an adequate comparable parallel group, making interpretation challenging [22].

Liraglutide is an antidiabetic agent that belongs to the class of medications known as GLP-1RAs. An antidiabetic medication that has a positive impact on indices related to HFpEF would be highly valuable in clinical practice. In the current meta-analysis, the notable decrease in E/Ea (a measure of left ventricular filling pressure) is an encouraging development. It has been previously demonstrated that elevated filling pressure independently predicts the progression of HFpEF in individuals with type 2 diabetes [26]. Possible underlying cardiac pathological mechanisms in the mentioned context include increased wall stress, diffuse cardiac fibrosis, and LV hypertrophy [27]. Liraglutide appears to have a positive impact on these pathological pathways, as evidenced by a decrease in the E/Ea ratio and also a tendency toward improved LV compliance and NTproBNP (N-terminal prohormone of brain natriuretic peptide) levels when compared to a placebo in previous studies [28]. Consequently, it is conceivable that initiating liraglutide treatment during the early asymptomatic stage of diabetic cardiomyopathy may delay the onset of clinically significant HFpEF. Regarding systolic function, we propose that the reduced LV filling volume directly leads to decreased stroke volume and ejection fraction. The slight decrease in ejection fraction observed in this meta-analysis is not deemed clinically significant as it remained within the normal range [28]. Additionally, there were no significant changes in cardiac output and cardiac index, likely due to the documented increase in heart rate associated with GLP-1RA therapy [9].

The European Association for the Study of Diabetes and the European Society of Cardiology recently collaborated to establish guidelines that make reference to CV outcome trials of glucose-lowering treatments and offer recommendations based on their results [9]. The recommendations acknowledge that GLP-1RAs (liraglutide, semaglutide, lixisenatide, exenatide, and dulaglutide) may be used to treat diabetes in patients with heart failure because their placebo-controlled randomized trials revealed no difference in the risk of HF hospitalization [9]. In order to lower CV events and the risk of death, liraglutide is also advised for patients with type 2 diabetes and CV disease or extremely high/high CV risk [29].

According to this study, liraglutide significantly affects LV diastolic function. This study demonstrates that liraglutide administration for a brief period is safe in individuals with type 2 diabetes, LV diastolic insufficiency, and no signs of heart failure. We argue against routinely evaluating heart function with imaging in these patients because there are now no clinical implications for the specific patient. It is significant to highlight that the majority of the studies included in this meta-analysis excluded HFpEF patients who had New York Heart Association classes III or IV [28]. As these patients have higher E/Ea, the effects of liraglutide therapy in this population cannot be extrapolated from our study. Liraglutide may even raise the likelihood of aggravation of heart failure symptoms and decompensation in this particular subgroup of patients since they are dependent on higher E/Ea for appropriate LV filling [27].

Study limitations

Despite the valuable findings obtained in this study, there are still some limitations to consider. Firstly, in our meta-analysis, we did not examine the impact of other primary drug treatments, such as spironolactone, which may influence cardiac function. Secondly, although ventricular diastolic function showed improvement, the majority of studies did not include patients with a history of heart failure. As a result, we were unable to explore the specific effects of the drug on patients with a history of heart failure. Moreover, due to a lack of individual-level data, we were unable to perform effect of different individual covariates on cardiac function. Lastly, the sample size of the included studies was low, and only one study assessed the long-term effect of liraglutide at 52 weeks. Therefore, long-term impacts of liraglutide were not assessed. Therefore, in the future, further large-scale RCTs are needed to assess long-term efficacy of liraglutide in type 2 diabetes patients.

## Conclusions

In this meta-analysis, we examined the effect of liraglutide on cardiac function in individuals with type 2 diabetes. The results indicated that liraglutide significantly reduced the E/A ratio and the E/Ea ratio, suggesting a potential clinical benefit on ventricular diastolic function. However, there was no significant impact on LVEF, cardiac output, cardiac index, and stroke volume, indicating that liraglutide did not improve systolic function. Further research is needed to fully understand the impact of liraglutide on different aspects of cardiac function in patients with type 2 diabetes, particularly in subgroups with heart failure. Despite these limitations, this study supports the potential of liraglutide in improving diastolic function and highlights its role as a valuable treatment option for individuals with type 2 diabetes.

## Appendices

PubMed

"Diabetes Mellitus, Type 2"[Mesh] OR "Type 2 diabetes" [tw] OR “T2DM” [tw] OR “diabetes” [tw] AND "Liraglutide" [tw] OR “victoza” [tw] OR “saxenda”[tw] AND “cardiac function” [tw] OR “diastolic cardiac function” [tw] OR “E to A ratio” [tw] OR “E/A” [tw] OR “E to Ea ratio” [tw] OR “E/Ea” [tw] OR “systolic cardiac function” [tw] OR “left ventricular ejection fraction” [tw] OR “LVEF” [tw] OR “ejection fraction” [tw] OR “stroke volume” [tw] OR “cardiac output” [tw] OR “cardiac index” [tw]

Cochrane Library

#1           MeSH descriptor: [Diabetes Mellitus, Type 2] explode all trees

#2           ("type 2 diabetes mellitus"):ti,ab,kw OR (type 2 diabetes):ti,ab,kw OR (T2DM):ti,ab,kw OR (diabetes):ti,ab,kw (Word variations have been searched) 

#3           (liraglutide):ti,ab,kw OR (victoza):ti,ab,kw OR (saxenda):ti,ab,kw (Word variations have been searched)

#4 (“cardiac function”): ti,ab,kw OR (diastolic cardiac function):ti,ab,kw OR (E to A ratio): ti,ab,kw OR (E/A):ti,ab,kw OR (E to Ea ratio):ti,ab,kw OR (E/Ea):ti,ab,kw OR (systolic cardiac function):ti,ab,kw OR (left ventricular ejection fraction):ti,ab,kw OR (LVEF):ti,ab,kw OR (ejection fraction):ti,ab,kw OR (stroke volume):ti,ab,kw AND (cardiac output):ti,ab,kw OR (cardiac index):ti,ab,kw (Word variations have been searched)

#1 AND #2 AND #3 AND #4

ti,ab,kw = terms in either title or abstract or keyword fields
